# Does the positive association between social relationships and cognition continue until very old age?

**DOI:** 10.1007/s10433-024-00835-9

**Published:** 2024-12-12

**Authors:** Selina Vogel, Andrés Oliva y Hausmann, Susanne Zank

**Affiliations:** 1 Department of Rehabilitation and Special Education, University of Cologne, Cologne, Germany; 2 Centre for Curative Gerontology, University of Cologne, Cologne, Germany

**Keywords:** Cognition, Episodic memory, Social relationships, Social convoys, Very old age, Prevention

## Abstract

In current research, social relationships are increasingly recognized for their positive associations with cognitive outcomes in older adults. One of the most vulnerable groups for cognitive decline are very old adults (80+ years). However, they are relatively underrepresented in the field. Therefore, our study aimed to test if social relationships remain a protective factor against cognitive decline in very old age, using a representative sample from the Study of Quality of Life and Well-Being in North-Rhine Westphalia (NRW80+ Study). We hypothesized that social characteristics would be positively associated with global cognition and episodic memory cross-sectionally and would predict cognitive performance two years later. 1.207 very old adults were included in the representative, cross-sectional analyses, and 639 in the panel analyses. They were aged between 80 and 103 years and showed no signs of dementia. The associations between various social aspects and cognitive functions were investigated using hierarchical linear regression, controlling for relevant sociodemographic and health characteristics. Cross-sectionally, leisure engagement was positively associated with episodic memory (*β* = 0.53 [0.26, 0.79], *p* < .01) and global cognition (*β* = 0.50 [0.22, 0.79], *p* < .01), while overall network size was positively associated with global cognition (*β* = 0.04 [0.02, 0.07], *p* < .01). In contrast, we observed no associations between baseline social relationships and cognitive functions two years later. The findings suggest that while social relationships are associated with cognitive functions in very old age, short-term protective effects such as over two years may be less robust compared to other age groups.

**Supplementary Information:**

The online version contains supplementary material available at 10.1007/s10433-024-00835-9.

## Introduction

A major desire of older adults is to maintain their independence for as long as possible. This independence is often compromised by age-related health risks, particularly in very old age (80+ years) (Brijoux et al. 2021). Cognitive impairment is an age-dependent health condition that severely impacts individual autonomy, as even mild cognitive impairment has noticeable consequences for daily living (Hussennoeder et al. 2020; Pais et al. 2020). Approximately one-third of individuals aged 80 years and above are affected by cognitive impairment (Gardner et al. 2013; Yaffe et al. 2011). It explains why cognitive decline is one of the most common health concerns of older adults (Malani et al. 2019; Murman 2015).

Particularly, episodic memory decline represents a persistent worry (Malani et al. 2019; Molden and Maxfield 2017). It involves encoding, storing, and recalling personal experiences and thereby is essential for maintaining an independent lifestyle (Duarte and Dulas 2020). Due to its sensitivity to normal and pathological aging, episodic memory has gained increased attention in cognitive research (McFall et al. 2019; Salthouse 2019). There is high variability within individual memory trajectories in both non-pathological and pathological aging (James et al. 2023; Salthouse 2019). It underlines that the decline cannot be solely attributed to aging. This encouraged research to find lifestyle factors that explain individual cognitive trajectories.

### Social relationships over the lifespan

Social relationships are a complex construct. The Convoy Model of Social Relations describes them as a dynamic network of individuals with varying levels of intimacy (Antonucci et al. 2014). They are influenced by life events like childbirth or retirement and thus, change over the lifetime. While research emphasizes their age-dependent dynamics, it also underlines that close relationships such as family or spouses show more stability over the lifetime than distant connections with acquaintances (Antonucci et al. 2019; Fuller et al. 2020). Furthermore, when studying social relationships, it is crucial to acknowledge their various dimensions. The structure of social relationships refers to the existence of social contacts by measures such as network size or contact frequency. The functional dimension describes processes within these networks, such as received emotional and instrumental support. Lastly, qualitative aspects focus on positive and negative aspects of social relationships such as marital satisfaction or relationship strain.

Particularly in very old age, social networks tend to change. Structurally, particularly more distant ties tend to lessen because chronic diseases, mobility issues, and frailty may limit the ability of social engagement (Adams et al. 2011; Freedman and Nicolle 2020; Fuller et al. 2020; Paine et al. 2022). Likewise, close relationships may decrease due to the loss of a spouse or a close peer or their move into long-term care facilities (d'Epinay et al. 2010). Despite those challenges, very old adults tend to report higher satisfaction with their social networks than younger adults (Rook and Charles 2017). This observation is captured by the socioemotional selectivity theory, positing that our social goals change with increasing age (Carstensen 2006). Older adults seem to prioritize emotional goals in relationships, focusing on fulfilling relationships while avoiding negative social interactions (Luong et al. 2011). Additionally, as proposed by the social input model, the behavioral change following the emotional shifts of older adults is reciprocated by their social partners, further reinforcing positive relationships (Fingerman and Charles 2010). To conclude, social relationships represent a multifaceted construct, whose perception, experience, and influence change over the lifespan.

### Social relationships and cognitive functions

Among others, social relationships have been proposed to positively affect cognition in old age in comparable effect sizes to other lifestyle factors such as physical activity (Bourassa et al. 2017). Indicators of good social relationships, such as having a confidant or frequent social contact, have been associated with higher cognitive performance in cross-sectional and longitudinal studies (Kuiper et al. 2016; Piolatto et al. 2022; Samtani et al. 2022). Particularly, structural aspects such as a large network and high activity level have consistently been linked to higher global cognition and episodic memory in older age (Evans et al. 2019; Piolatto et al. 2022). These findings are attributed to their potential to increase cognitive reserve. Cognitive reserve defines an ability to buffer against the consequences of pathological brain processes, explaining why some individuals with brain pathology do not develop cognitive symptoms (Sachdev 2022). Specifically, social relationships are assumed to reinforce cognitive reserve through mental stimulation, stress-relieving effects, and increased health access (Kuiper et al. 2016). Aligning with the "use-it-or-lose-it" principle, social engagement stimulates cognitive processes and helps to maintain these functions. Supportive and positive relationships also reduce stress and its harmful effects such as high cortisol or inflammation, which influence brain functions. Additionally, social ties encourage healthy behaviors like physical activity, a nutritious diet, or medical check-ups (Sachdev 2022). Good social relationships may particularly benefit memory because the underlying brain regions appear very sensitive to those mechanisms as they are very neuroplastic and show high sensitivity to stress-related physiological effects (Bartsch and Wulff 2015; Kim et al. 2015).

Still, there are some unanswered questions. For instance, it is unclear whether the positive effects of social relationships on cognition persist until very old age (80+ years). Vos et al. (2017) suggested that the influence of lifestyle factors decreases with advancing age. In their study, factors like smoking, low education, or physical inactivity showed little to no association with the risk of dementia in very old compared to younger adults. It could be due to the growing impact of brain pathology with age, which may limit the scope of cognitive reserve. Also, survival biases may explain the weakened effect: Those who reach very old age without cognitive impairment, despite lifestyle risks like physical inactivity or social isolation, may have other protective traits like genetic dispositions that compensate for these risks. To conclude, further research is needed to understand the dynamics of social relationships and cognition across various ages, particularly in the presence of increased health constraints and multimorbidity, such as in very old age. So far, research on very old age is limited to a few studies that show partly disagreeing results (Grothe et al. 2022; Huang et al. 2023; Röhr et al. 2020).

### The present study

In summary, cognitive functioning is essential for maintaining an active lifestyle (Hussennoeder et al. 2020). Very old adults have the highest risk of cognitive impairment compared to other younger ages, underlining the importance of finding preventive approaches in this group (Gardner et al. 2013). Good social relationships have been linked to better cognitive outcomes in older age, but most studies have focused on young-old age (65+ years) (Piolatto et al. 2022). For very old age, some evidence suggests that the impact of social aspects may decrease (Grothe et al. 2022; Vos et al. 2017).

Hence, the present study aims to investigate the associations between social connections and cognitive functions in very old age. We focus on global cognition as a comprehensive cognitive measure and episodic memory as a more change-sensitive measure (McFall et al. 2019; Salthouse 2019). As research particularly highlights the positive association between the structure of social relationships and cognitive functions, our study mainly focuses on structural social aspects. Specifically, we incorporate information on close and distant social networks, following the convoy approach (Fuller et al. 2020). We hypothesize that the social aspects will be positively associated with cognitive functions cross-sectionally and predict cognitive performance two years later (Huang et al. 2023; Röhr et al. 2020). The hypothesis is based on the theory that social relationships enhance cognitive reserve by providing mental stimulation, health access, and stress reduction. Structural aspects of social relationships are particularly assumed to operate through their cognitive enrichment (Sachdev 2022).

## Method

### Study design

The present study used data from the Study of Quality of Life and Well-Being in North-Rhine Westphalia (NRW80+ Study). The de-identified data is available at the GESIS—Leibniz Institute for the Social Sciences data repository (Albrecht et al. 2022; Zank et al. 2022). The NRW80+ Study investigated the health, well-being, and quality of life of very old adults (80+ years) from North-Rhine-Westphalia (NRW) with two measurement waves. NRW represents the largest German state, reflecting the diversity of entire Germany by its proximity of rural and urban areas and its immigration history. Sampling was conducted through a two-step method, by which participants were randomly chosen from 94 selected communities of NRW (Hansen et al. 2021). It included residents from private and stationary households. The assessments were administered as computer-assisted personal or proxy interviews. Proxy interviews were conducted if participants were unable to answer personally but consented that a trusted person answers on their behalf. During the first wave from 2017 to 2018 (*T*_1_), 1.863 very old adults were assessed. In the second measurement wave from 2019 to 2021 (*T*_2_), 1.612 participants were consentingly re-contacted, with 912 receiving a second assessment.

### Participants

**Fig. 1:**
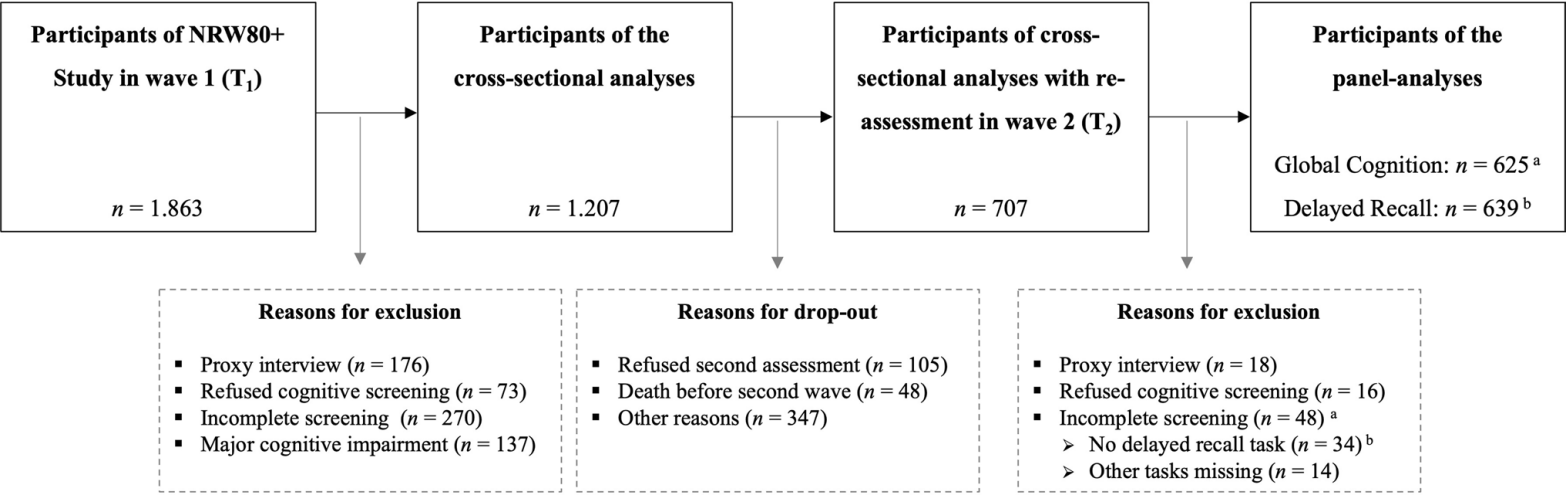
Flowchart of the sample selection process. Other reasons for dropping out in wave 2 included health constraints, address- or scheduling issues, and termination of assessments

The present study focused on participants from the first wave of the NRW80+ Study, including participants with a single assessment and a re-assessment in the second wave. Specifically, we focused on participants with no indication of dementia at the first wave to minimize the possibility of reverse causality. Probable dementia was indicated by the total score on the Dementia Detection Test (DemTect), a sensitive screening for cognitive impairment (Kalbe et al. 2004). Its score ranged from 0 to 18 points: 13–18 points represented an age-appropriate performance, 9–12 points suggested mild cognitive impairment (MCI), and scores below 9 indicated probable dementia (Kessler et al. 2014). Participants without a DemTect total score—those who refused the assessment, had incomplete data, or were rated by a proxy—were also excluded. The final sample included 1.207 participants for the cross-sectional analyses. From the panel sample, 639 participants were used for the memory analyses and 625 for global cognition. Further details are displayed in Fig. 1.

### Research materials

#### Outcome variables: Episodic memory and global cognition

In the present study, cognition was measured by the DemTect (Kalbe et al. 2004). Compared to the Mini-Mental-State-Examination (MMSE), it demonstrates a higher sensitivity for minor cognitive impairment (Kalbe et al. 2005). It comprises five cognitive tasks—immediate recall, number transcoding, verbal fluency, digit span, and delayed memory recall. In the immediate recall, participants recall a list of 10 words twice after hearing it. For number transcoding, they convert numbers into numerals and vice versa (example: 209 to two hundred nine; six hundred eighty-one to 681). During verbal fluency, they name as many supermarket products as possible within one minute. In the digit backward span, they repeat number sequences in reverse order. In the delayed recall, they freely recall the wordlist of the first task after approximately 10 min. We used the delayed recall performance to measure episodic memory, ranging from 0 to 10 points. Furthermore, the total score of the DemTect was used to indicate global cognition. It ranged from 0 to 18, summarizing the age-standardized performance of all five subtasks.

#### Predictor variables: Structural social aspects

Our study focused on the (primarily) structural social aspects of the NRW80+ study, encompassing five key variables. Marital status was coded as 1 (“single or divorced”), 2 (“widowed”), or 3 (“married or partnered”). The close network size was derived from participants naming their four most important social ties and rating their connection to them on a scale from 1 (“not close at all”) to 4 (“very close”). These ratings were recoded as 0 (“no or less close person”), 1 (“close person”), and 2 (“very close person”). The total score ranged from 0 to 8. McDonald's Omega was acceptable in the cross-sectional sample (*ω* = 0.78) and the panel sample (*T*_1_ *ω* = 0.77). Overall network size was assessed by asking participants to indicate their total number of social contacts. The score ranged from 0 ("no contacts") to 30 ("30 or more contacts"). Contact frequency was included as a mean score of interaction frequency with the four most important ties. For each member, interaction frequency was rated from 1 ("less than yearly") to 5 ("daily"). McDonald's Omega was acceptable in the cross-sectional sample (*ω* = 0.76) and the panel sample (*T*_1_ *ω* = 0.75). Lastly, leisure engagement was indicated by the mean frequency of engagement in 14 leisure activities, which were rated from 0 ("never") to 5 ("daily"). The list of all activities is available in the supplementary materials. McDonald's Omega was (minimally) acceptable in the cross-sectional sample (*ω* = 0.63) and in the panel sample (*T*_1_ *ω* = 0.62).

#### Further control variables

*Demographics*. The age of participants was indicated as a continuous variable with two decimals. Sex was assigned as 1 (“male”) or 2 (“female”). Education was classified as 1 (“low”), 2 (“medium”), or 3 (“high”). The form of housing was indicated by 1 (“private”) or 2 (“stationary”).

*Social variables*. Research shows that while social networks become smaller with age, the satisfaction with them does not change equally. To control for this, we included loneliness as a confounding variable. It was assessed with a single item rating feelings of loneliness over the last two weeks from 1 ("never") to 4 ("always").

*Physical and mental health*. Mental and physical health represent potential confounders in the association between social relationships and cognition. Thus, we accounted for functional ability by asking participants to rate their independence on seven instrumental activities of daily living from 0 ("with full help") to 2 ("without help"). McDonald's Omega was good in the cross-sectional sample (*ω* = 0.88) and the panel sample (*T*_1_ *ω* = 0.88). For the panel analyses, the time in months between *T*_1_ and *T*_2_ was included as a continuous variable. Furthermore, depressiveness was measured with the short version of the Depression in Age Scale (DIA-S4), a screening tool comprising four yes/no items on the presence of various depressive symptoms over the last two weeks (Heidenblut and Zank 2010). McDonald's Omega was acceptable in the cross-sectional sample (*ω* = 0.75) and minimally acceptable in the panel sample (*T*_1_ *ω* = 0.60).

### Statistical analyses

The data was prepared in IBM SPSS. The main analyses were performed with the standard R stats package (Core Team 2023). Furthermore, we used the "mice" package to impute missing data in the predictor variables (van Buuren and Groothuis-Oudshoorn 2011). We did not impute the dependent variables because they did not meet the missing at random (MAR) assumption. Overall, 121 cross-sectional and 46 panel participants showed missing values in the predictors, but no variable had more than 5% missingness. We generated 20 imputed samples using predictive mean matching (PMM), which selects a data point from the non-missing data with predicted values close to those of the missing sample.

We used hierarchical linear regression to answer our hypotheses. Likelihood-ratio tests determined if the model fit improved when further predictors were added to the baseline models. To avoid biases arising from sample recruitment, our analyses were weighted. In the cross-sectional sample, the weights adjusted for selection probability through age, sex, household size, regional area and type, municipality size, form of housing, and marital status. In the panel sample, the weights further controlled for relevant *T*_1_ risk factors of *T*_2_ dropout such as older age, lower socioeconomic status, lower cognition, lower health, smaller households, and decreased urbanization (Wagner and Zank 2022). In all analyses, we used a reversed Helmert contrast for the ordinal variables "education" and "marital status" and a Helmert contrast for “loneliness”.

In the cross-sectional analyses, we aimed to investigate if social relationships were associated with cognitive performance throughout very old age. The baseline models included cognitive performance (global cognition or delayed recall) as outcomes and age, sex, education, and housing form as predictors (model 1). Subsequent models added the structural social aspects (marital status, close network size, overall network size, contact frequency, leisure activity) (model 2), and further control variables (loneliness, functional ability, depressive symptoms) (model 3). In the panel analyses, the aim was to investigate if social relationships predicted cognitive change two years later. The baseline model included *T*_2_ cognitive performance (global cognition or delayed recall) as outcomes and *T*_1_ cognitive performance, *T*_1_ age, sex, education, *T*_1_ housing form, and the time between measurements as predictors (model 1). Subsequent models added *T*_1_ the structural social aspects (model 2), and *T*_1_ control variables (model 3).

#### Sensitivity analyses

To ensure the robustness of the results, we conducted the analyses without survey weights, imputation, and participants with MCI.

## Results

### Sample characteristics

The sample characteristics of the imputed cross-sectional sample and panel sample are provided in Table 1. Bivariate correlations of all included variables are available in the supplementary materials (Tables S1 and S2).

**Table 1:** Descriptive statistics of the unweighted and weighted cross-sectional and panel sample

Predictors	Means (SD) and frequencies			
	Cross-sectional sample (*n* = 1207^a^)		Panel sample (*n* = 639^b^)	
	Unweighted	Weighted	Unweighted	Weighted
*T*_*1*_* variables*				
Age	86.20 (4.25)	84.87 (3.70)	85.80 (4.07)	84.92 (3.81)
Male sex	51.30%	36.60%	51.80%	37.20%
Education				
Low education	21.90%	25.90%	19.10%	23.90%
Medium education	52.40%	51.30%	51.20%	50.50%
Private housing	95.90%	94.40%	95.60%	92.00%
Marital status				
Single or divorced	6.40%	8.60%	6.40%	9.20%
Widowed	48.50%	48.90%	49.00%	48.30%
Close network size	5.12 (2.15)	5.13 (2.17)	5.21 (2.09)	5.14 (2.12)
Overall network size	7.65 (6.75)	7.57 (6.70)	7.85 (6.32)	7.35 (6.03)
Contact frequency	3.49 (1.15)	3.48 (1.16)	3.54 (1.09)	3.47 (1.12)
Leisure engagement	1.39 (0.61)	1.45 (0.60)	1.51 (0.60)	1.52 (0.60)
Loneliness				
Never	77.50%	78.60%	79.00%	79.90%
Sometimes	17.70%	17.50%	17.10%	16.20%
Often	2.80%	1.90%	2.30%	1.70%
Functional ability	1.61 (0.50)	1.63 (0.49)	1.69 (0.45)	1.65 (0.49)
Depressive symptoms	0.79 (1.03)	0.79 (1.04)	0.68 (0.98)	0.74 (1.02)
Delayed recall	4.38 (2.39)	4.53 (2.43)	4.67 (2.34)	4.66 (2.43)
Global cognition	15.20 (2.69)	15.36 (2.70)	15.70 (2.52)	15.49 (2.71)
*T*_*2*_* variables*				
Delayed recall	–	–	4.15 (2.46)	4.26 (2.58)
Global cognition	–	–	14.90 (3.36)	14.96 (3.39)
Months between *T*_1_ and *T*_2_	–	–	22.20 (2.35)	22.29 (2.39)

^a^*n* = 121 had missing values in at least one and at most four predictors. Variables with > 2% missingness included depressive symptoms (4.7%) and education (3.8%)

^b^*n* = 46 had missing values in at least one and at most three predictors. Variables with > 2% missingness included depressive symptoms (4.8%) and education (2.2%). The outcomes were not imputed, *n* = 14 had no global cognition score

### Results of the regression analyses

We exclusively report the results of the model comparisons and the final, fully adjusted models. The final models of the cross-sectional and panel analyses are shown in Tables 2 and 3, respectively.

**Table 2:** Regression models examining delayed recall and global cognition in the cross-sectional sample

Predictors	Delayed memory	Global cognition
	Estimate (95% CI)	Estimate (95% CI)
Intercept	7.67 (4.05, 11.30)^**^	15.66 (11.76, 19.56)^**^
Age	− 0.06 (− 0.10, − 0.02)^**^	− 0.03 (− 0.08, 0.01)
Female sex *(ref. male sex)*	0.50 (0.16, 0.83)^**^	0.69 (0.32, 1.05)^**^
Education		
Medium* (ref. low)*	0.09 (− 0.09, 0.26)	0.28 (0.09, 0.48)^**^
High* (ref. low/medium)*	0.14 (0.01, 0.26)^*^	0.32 (0.19, 0.46)^**^
Stationary housing (*ref. private housing*)	− 0.25 (− 0.89, 0.39)	− 1.09 (− 1.78, − 0.41)^**^
Marital status		
Widowed* (ref. single/divorced)*	0.04 (− 0.21, 0.30)	0.23 (− 0.04, 0.51)
Married/partnered* (ref. single/divorced/widowed)*	− 0.06 (− 0.18, 0.07)	0.02 (− 0.11, 0.15)
Close network size	0.03 (− 0.08, 0.13)	0.01 (− 0.10, 0.12)
Overall network size	0.02 (− 0.01, 0.04)	0.04 (0.02–0.07)^**^
Contact frequency	− 0.03 (− 0.22, 0.17)	− 0.16 (− 0.37, 0.05)
Leisure engagement	0.53 (0.26, 0.79)^**^	0.50 (0.22, 0.79)^**^
Loneliness		
Never* (ref. sometimes/often/always)*	0.02 (− 0.49, 0.53)	0.03 (− 0.52, 0.58)
Sometimes* (ref. often/always)*	− 0.17 (− 0.91, 0.57)	0.22 (− 0.58, 1.01)
Often* (ref. always)*	− 0.20 (− 1.52, 1.12)	− 0.89 (− 2.32, 0.53)
Functional ability	0.40 (− 0.06, 0.73)^*^	0.85 (0.49, 1.22)^**^
Depressive symptoms	0.13 (− 0.02, 0.28)	0.15 (0.01, 0.31)
*R*-Squared	0.07	0.13
*N*	1207	1207

*p ≤ .05, **p ≤ .01

**Table 3:** Regression models examining delayed recall and global cognition in the panel sample

Predictors	^T2^Delayed memory	^T2^Global cognition
	Estimate (95% CI)	Estimate (95% CI)
Intercept	3.46 (− 1.26, 8.17)	8.20 (0.68, 13.48)^*^
^T1^Performance	0.56 (0.50, 0.63)^**^	0.66 (0.57, 0.75)^**^
Months between *T*_1_ and *T*_2_	0.02 (− 0.04, 0.09)	0.10 (0.01, 0.19)^*^
^T1^Age	− 0.05 (− 0.10, 0.00)^*^	− 0.08 (− 0.14, − 0.01)^*^
Male sex	0.40 (− 0.01, 0.81)	0.28 (− 0.26, 0.82)
Education		
Medium* (ref. low)*	− 0.06 (− 0.28, 0.16)	− 0.11 (− 0.40, 0.18)
High* (ref. low/medium)*	0.01 (− 0.13, 0.16)	− 0.01 (− 0.20, 0.18)
^T1^Private housing	− 1.00 (− 1.68, − 0.32)^**^	− 0.68 (− 1.60–0.25)
^T1^Marital status		
Widowed* (ref. single/divorced)*	− 0.10 (− 0.41, 0.21)	− 0.18 (− 0.60, 0.24)
Married/partnered* (ref. single/divorced/widowed)*	− 0.07 (− 0.22, 0.08)	− 0.01 (− 0.21, 0.19)
^T1^Close network size	− 0.01 (− 0.14, 0.11)	− 0.12 (− 0.28, 0.04)
^T1^Overall network size	0.00 (− 0.03, 0.03)	0.03 (− 0.01, 0.07)
^T1^Contact frequency	− 0.04 (− 0.27, 0.20)	0.00 (− 0.31, 0.31)
^T1^Leisure engagement	0.24 (− 0.09, 0.57)	0.29 (− 0.14, 0.73)
^T1^Loneliness		
Never* (ref. sometimes/often/always)*	0.01 (− 0.60, 0.59)	0.29 (− 0.49, 1.07)
Sometimes* (ref. often/always)*	− 0.14 (− 1.02, 0.74)	0.26 (− 0.90, 1.43)
Often* (ref. always)*	0.08 (− 1.52, 1.68)	− 0.19 (− 2.31, 1.92)
^T1^Functional ability	0.76 (0.34, 1.18)^**^	0.87 (0.29, 1.44)^**^
^T1^Depressive symptoms	0.19 (− 0.01, 0.39)	0.12 (− 0.12, 0.37)
*R*-Squared	0.41	0.41
*N*	639	625

*p ≤ .05, **p ≤ .01

#### Cross-sectional analyses

*Delayed recall*. A significant improvement in model fit was observed from model 1 to model 2 (*F*(6, 1192) = 5.63, *p* < 0.01), i.e., when structural social aspects were added. No improvement was observed from model 2 to model 3 (*F*(5, 1880) = 1.65, *p* = 0.14), when, i.e., loneliness, depressive symptoms, and functional ability were added. The final model accounted for 7.22% of the variance in delayed recall. Younger age (*β* = − 0.06 [− 0.10, − 0.02], *p* < 0.01), female compared to male sex (*β* = 0.50 [0.16, 0.83], *p* < 0.01), high compared to low and moderate education (*β* = 0.14 [0.01, 0.26], *p* = 0.03), more leisure engagement (*β* = 0.53 [0.26, 0.79], *p* < 0.01) and higher functional ability (*β* = 0.40 [0.06, 0.73], *p* = 0.02) were associated with higher delayed recall. There were no associations with other predictors.

*Global cognition*. A significant improvement in model fit was observed from model 1 to model 2 (*F*(6, 1191) = 7.80, *p* < 0.01) and from model 2 to model 3 (*F*(5, 1885) = 5.12, *p* < 0.01). The final model accounted for 12.77% of the variance in global cognition. Female compared to male sex (*β* = 0.69 [0.32, 1.05], *p* < 0.01), moderate compared to low education (*β* = 0.28 [0.09, 0.48], *p* < 0.01), high compared to low and moderate education (*β* = 0.32 [0.19, 0.46], *p* < 0.01), living in private housing (*β* = 1.09 [− 1.78, − 0.41], *p* < 0.01), a larger social network (*β* = 0.04 [0.02, 0.07], *p* < 0.01), more leisure engagement (*β* = 0.50 [0.22, 0.79], *p* < 0.01) and higher functional ability (*β* = 0.85 [0.49, 1.22], *p* < 0.01) were associated with higher global cognition. There were no associations with other predictors.

#### Panel analyses

*Delayed recall*. A significant improvement in model fit was observed from model 2 to model 3 (*F*(5, 615) = 3.08, *p* = < 0.01), when loneliness, depressive symptoms, and functional ability were added. No improvement was observed from model 1 to model 2 (*F*(6, 623) = 1.38, *p* = 0.22), when structural social aspects were added. The final model accounted for 40.58% of the variance in delayed recall performance. Higher *T*_1_ cognitive performance (*β* = 0.56 [0.50, 0.63], *p* < 0.00), younger *T*_1_ age (*β* = − 0.05 [− 0.10, 0.00], *p* = 0.05), female compared to male sex (*β* = 0.40 [− 0.01, 0.81], *p* = 0.01), private housing in *T*_1_ (*β* = − 1.00 [− 1.68, − 0.32], *p* < 0.00), and higher *T*_1_ functional ability (*β* = 0.76 [0.34, 1.18], *p* < 0.00) were associated with higher delayed recall at *T*_2_. There were no associations with other predictors.

*Global cognition*. A significant improvement in model fit was observed from model 1 to model 2 (*F*(6, 609) = 2.06, *p* = 0.05). No improvement was observed from model 2 to model 3 (*F*(5, 602) = 1.97, *p* = 0.08). The final model accounted for 40.64% of the variance in global cognition performance. Higher *T*_1_ cognitive performance (*β* = 0.66 [0.57, 0.75], *p* < 0.01), younger *T*_1_ age (*β* = − 0.08 [− 0.14, − 0.01], *p* = 0.02), and higher *T*_1_ functional ability (*β* = 0.87 [0.29, 1.44], *p* < 0.01) were associated with higher global cognition at *T*_2_. Furthermore, a longer time interval between the assessments was associated with a higher global cognition at *T*_2_ (*β* = 0.10 [0.01, 0.19], *p* = 0.03). There were no associations with other predictors.

### Results of the sensitivity analyses

Results of the sensitivity analyses are provided in the supplementary materials (Tables S3 and S8). Overall, outcomes were mainly consistent when models were calculated without weights, imputation, and participants with MCI.

## Discussion

The present study examined the associations between social relationships and cognition in very old adults cross-sectionally and over approximately two years. Cognition was assessed using measures of global cognition and delayed memory, while social relationships encompassed structural aspects of both close and more distant convoys. We hypothesized that social aspects would be positively associated with cognitive functions cross-sectionally and would predict cognitive performance two years later. We used hierarchical regression to investigate the associations between social aspects and cognitive performance, accounting for relevant demographic, social, and health characteristics. Our sample consisted of 1207 participants from the NRW80+ Study. They were aged between 80 and 103 years and showed no signs of dementia. From them, 639 participants were reassessed after approximately two years. We found partial evidence for our hypothesis. In the cross-sectional analyses, social network size and leisure engagement were positively associated with global cognition, while more leisure engagement was also associated with better episodic memory performance. However, social aspects did not predict cognitive performance two years later.

Consistent with previous research, our cross-sectional analyses showed a positive association between leisure engagement, overall network size, and cognitive functions, aligning with the findings of previous studies (Huang et al. 2023; Rodriguez et al. 2018; Röhr et al. 2020). Leisure engagement and total network size indicate valuable resources for mental stimulation and health access, thereby aiding cognitive reserve (Sachdev 2022). Meanwhile, no associations were observed between aspects of the close network including marital status, close network size, and contact frequency with the four most important social ties. This contradicts previous research suggesting that a high-quality network is positively associated with cognition in older age (Hülür 2022; Luo et al. 2021; Saito et al. 2021; Zahodne et al. 2019). Several explanations could account for the discrepancy. Importantly, other studies primarily addressed middle-aged and young-old adults. In very old age, social networks become smaller and more close-knit (Fuller et al. 2020). It was also evident in our samples, as participants showed a high proportion of close social ties and had very frequent contact with them. This may result in limited variability, explaining the lack of associations with cognitive functioning. Close social networks also become more family-centered with age (Fuller et al. 2020). Indeed, our participants showed a very high proportion of family members in their close networks. It suggests that interactions may involve more established routines and practices, which may limit cognitive enrichment (Sachdev 2022). The importance of heterogeneity in social relationships as a resource for cognitive stimulation and information gathering has been pointed out by multiple studies (Piolatto et al. 2022). Lastly, varying operationalizations of close networks may explain the discrepancies between findings. Studies have reported mixed results depending on how close social networks were operationalized, with some findings aligning with ours and others not (Fan et al. 2021; Perry et al. 2022; Hülür 2022; Zahodne et al. 2019). This also highlights the need for more standardized social measures as emphasized by recent meta-analyses (Kuiper et al. 2016; Piolatto et al. 2022). To conclude, the present study revealed that cognitive functions were linked to broader social aspects cross-sectionally, whereas it showed no associations with characteristics of close social relationships.

Furthermore, the social aspects did not predict cognitive functions two years later in the present study. This contradicts studies that have reported an association between social relationships and cognitive impairment over time in the oldest old (Huang et al. 2023; Röhr et al. 2020). The differences may be attributed to our shorter assessment period over approximately two years in contrast to the four-year periods in the other studies. Within two years, we mainly captured subtle cognitive changes. It could imply that social relationships may not have “fast-working” effects on cognition in very old age. This would align with the findings of Vos et al. (2017). They reported that the LIBRA score, which represents an individual lifestyle prevention potential against dementia, did not predict dementia in very old age as strongly as in midlife and young-old age (Vos et al. 2017). Interestingly, a study using the same dataset as Röhr et al. (2020), found no association between social isolation and global cognition over four years when controlling for survival time (Grothe et al. 2022). Indeed, the impact of lifestyle factors on cognition varies throughout the lifetime, and in very old age, increasing pathological processes related to lower health may lower the impact of lifestyle factors (Fratiglioni and Qiu 2011; Vos et al. 2017). Moreover, very old adults represent a selective group, possibly with certain genetic or other factors related to reaching such an old age that may partly compensate for lifestyle risks such as low social engagement. Another possible explanation for the discrepancies is, again, the different operationalization of social predictors. Previous studies on the oldest old often focused on social isolation as an overarching concept while we included multiple social aspects (Huang et al. 2023; Röhr et al. 2020). In sum, social relationships seem to contribute to cognition in very old age but do not seem to represent a quick-working preventive factor against cognitive decline.

## Limitations

Although the present study provides valuable insights, they should be interpreted with the following limitations. Firstly, the panel data consisted of only one repeated assessment two years later. As stated, this has limited the scope of cognitive changes to rather small and subtle alterations. Secondly, our study could not account for all confounding lifestyle factors, including smoking or alcohol consumption (Piolatto et al. 2022). Thirdly, our study primarily examined structural aspects, except for close network as a combination of structural and qualitative elements, and loneliness as a control variable for relationship satisfaction. We did not include functional measures like emotional or instrumental support, which may show other associations with cognition in very old age as they are more linked to stress-buffering mechanisms. Future research could further explore this direction in greater depth. Lastly, we acknowledge that the present associations may be underestimated due to sample selectivity. Although we accounted for possible biases using survey weights, there is a remaining risk of selectivity bias. It could underestimate phenomena like social isolation or cognitive decline and thereby underestimate our associations. Similarly, the decrease in sample size from the cross-sectional to the panel analyses may have affected the statistical power of the results.

## Conclusions

The present study explored the associations between social relationships and cognitive function in very old age, cross-sectionally and over two years. Specifically, we investigated if structural aspects of the close and broader social network are associated with global cognition and episodic memory in very old age, controlling for relevant sociodemographic and health factors. Our findings showed positive cross-sectional associations between overall network size and leisure engagement with cognitive functions. However, baseline social relationships did not predict cognitive functioning two years later. The results emphasize the lifelong associations between social relationships and cognition but also suggest that social relationships do not provide a “quick fix” against cognitive decline in very old age. Given the overall small effect sizes, they seem to represent a protective factor against cognitive decline, which may be more effective earlier in life as supported by other studies as well (Grothe et al. 2022; Vos et al. 2017). Finally, our results underline the importance of examining modifiable risk factors of cognitive decline across different life stages, as their impact likely varies with age (Fratiglioni and Qiu 2011).

## Supplementary Information

Below is the link to the electronic supplementary material.

Supplementary file1 (DOCX 56 KB)

## Data Availability

The present study used secondary data from the Study of Quality of Life and Well-Being in North-Rhine Westphalia (NRW80+ Study). The data is freely accessible through GESIS, Cologne. The cross-sectional data and related materials can be accessed at https://doi.org/10.4232/1.13978 and the panel data and its materials at https://doi.org/10.4232/1.13985.
