# Supplementary material for: Does the positive association between social relationships and cognition continue until very old age?
Source: Eur J Ageing. 2024 Dec 12;21(1):39. doi: 10.1007/s10433-024-00835-9 (PMC11635074; doi:10.1007/s10433-024-00835-9)
Supplement: Supplementary file 1 — Supplementary file1 (DOCX 56 KB) [file 10433_2024_835_MOESM1_ESM.docx]

European Journal of Ageing

**Does the positive association between social relationships and cognition continue until very old age?**

Selina Vogel^*1^ (<https://orcid.org/0000-0002-1814-2918>)

Andrés Oliva y Hausmann^1,2^ (<https://orcid.org/0000-0002-9605-1819>)

Susanne Zank^1,2^ (<https://orcid.org/0000-0002-8396-1610>)

^1^ Department of Rehabilitation and Special Education, University of Cologne, Germany

^2^ Centre for Curative Gerontology, University of Cologne, Germany

^*^Corresponding author: Selina Vogel, Department of Rehabilitation and Special Education, University of Cologne, North-Rhine-Westphalia, Germany. E-Mail: [svogel18@uni-koeln.de](mailto:svogel18@uni-koeln.de).

**Supplementary materials**

**Leisure engagement**

(1) Doing sports, (2) having tea parties, (3) visiting a café, (4) traveling, (5) visiting the cinema, (6) visiting a concert, theater, museum, (7) artistic activity, (8) volunteering, (9) playing board games, (10) continuing education, (11) joining a political event, (12) strolling, (13) hosting visitors, (14) participation in other hobby (specified if applicable)

**Table S1**. Bivariate correlations of the weighted cross-sectional sample (*n* = 1207).

|  | 1 | 2 | 3 | 4 | 5 | 6 | 7 | 8 | 9 | 10 | 11 | 12 | 13 |
| --- | --- | --- | --- | --- | --- | --- | --- | --- | --- | --- | --- | --- | --- |
| 1. Age |  |  |  |  |  |  |  |  |  |  |  |  |  |
| 2. Male Sex | .15^**^ |  |  |  |  |  |  |  |  |  |  |  |  |
| 3. Education | -.08^**^ | -.38^**^ |  |  |  |  |  |  |  |  |  |  |  |
| 4. Private Housing | .22^**^ | .11^**^ | -.11^**^ |  |  |  |  |  |  |  |  |  |  |
| 5. Marital Status | -.20^**^ | -.39^**^ | .12^**^ | -.13^**^ |  |  |  |  |  |  |  |  |  |
| 6. Close network size | -.03^**^ | .02^**^ | .02^*^ | -.07^**^ | .22^**^ |  |  |  |  |  |  |  |  |
| 7. Overall network size | -.04^**^ | .01 | .04^**^ | -.06^**^ | .10^**^ | .49^**^ |  |  |  |  |  |  |  |
| 8. Contact frequency | -.03^**^ | -.01 | .03^**^ | -.13^**^ | .22^**^ | .76^**^ | .51^**^ |  |  |  |  |  |  |
| 9. Leisure engagement | .10^**^ | .10^**^ | -.06^**^ | .08^**^ | -.18^**^ | -.18^**^ | -.19^**^ | -.15^**^ |  |  |  |  |  |
| 10. Loneliness | -.22^**^ | .01 | .25^**^ | -.06^**^ | .05^**^ | .14^**^ | .18^**^ | .27^**^ | -.18^**^ |  |  |  |  |
| 11. Functional ability | -.32^**^ | -.19^**^ | .23^**^ | -.34^**^ | .13^**^ | .02^**^ | .03^**^ | .08^**^ | -.18^**^ | .39^**^ |  |  |  |
| 12. Depressive symptoms | .07^**^ | .06^**^ | -.09^**^ | .11^**^ | -.03^**^ | -.11^**^ | -.07^**^ | -.08^**^ | .32^**^ | -.22^**^ | -.30^**^ |  |  |
| 13. Delayed recall | -.13^**^ | .06^**^ | .08^**^ | -.07^**^ | -.03^**^ | .05^**^ | .05^**^ | .10^**^ | -.04^**^ | .20^**^ | .14^**^ | -.02^**^ |  |
| 14. Global cognition | -.13^**^ | .01^*^ | .19^**^ | -.16^**^ | .01 | .03^**^ | .04^**^ | .12^**^ | -.03^**^ | .23^**^ | .24^**^ | -.04^**^ | .71^**^ |

*Note.* ^*^*p* ≤. 05. ^**^*p* ≤ .01.

**Table S2**. Bivariate correlations of the weighted panel sample (*n* = 639).

|  | 1 | 2 | 3 | 4 | 5 | 6 | 7 | 8 | 9 | 11 | 12 | 13 | 14 | 15 | 16 |
| --- | --- | --- | --- | --- | --- | --- | --- | --- | --- | --- | --- | --- | --- | --- | --- |
| 1. ^T1^ Age |  |  |  |  |  |  |  |  |  |  |  |  |  |  |  |
| 2. Male Sex | .18^**^ |  |  |  |  |  |  |  |  |  |  |  |  |  |  |
| 3. Education | -.07^**^ | -.42^**^ |  |  |  |  |  |  |  |  |  |  |  |  |  |
| 4. ^T1^ Private Housing | .26^**^ | .08^**^ | -.07^**^ |  |  |  |  |  |  |  |  |  |  |  |  |
| 5. ^T1^Marital Status | -.26^**^ | -.42^**^ | .11^**^ | -.20^**^ |  |  |  |  |  |  |  |  |  |  |  |
| 6. ^T1^ Close network size | -.08^**^ | -.02^*^ | -.01 | -.11^**^ | .26^**^ |  |  |  |  |  |  |  |  |  |  |
| 7. ^T1^ Overall network size | -.11^**^ | -.04^**^ | .03^**^ | -.15^**^ | .15^**^ | .52^**^ |  |  |  |  |  |  |  |  |  |
| 8. ^T1^ Contact frequency | -.10^**^ | -.06^**^ | .03^**^ | -.17^**^ | .24^**^ | .76^**^ | .53^**^ |  |  |  |  |  |  |  |  |
| 9. ^T1^ Leisure engagement | -.27^**^ | -.07^**^ | .21^**^ | -.11^**^ | .13^**^ | .14^**^ | .19^**^ | .24^**^ |  |  |  |  |  |  |  |
| 10. ^T1^ Loneliness | .14^**^ | .09^**^ | -.06^**^ | .08^**^ | -.17^**^ | -.15^**^ | -.18^**^ | -.10^**^ | -.22^**^ |  |  |  |  |  |  |
| 11. ^T1^ Functional ability | -.35^**^ | -.17^**^ | .19^**^ | -.40^**^ | .17^**^ | .09^**^ | .07^**^ | .13^**^ | .44^**^ |  |  |  |  |  |  |
| 12. ^T1^Depressive symptoms | .08^**^ | .10^**^ | -.09^**^ | .07^**^ | -.07^**^ | -.14^**^ | -.10^**^ | -.11^**^ | -.29^**^ | -.29^**^ |  |  |  |  |  |
| 13. ^T1^ Delayed recall | -.13^**^ | .13^**^ | .05^**^ | -.14^**^ | -.01 | .08^**^ | .03^**^ | .06^**^ | .20^**^ | .17^**^ | -.03^**^ |  |  |  |  |
| 14. ^T1^ Global cognition | -.12^**^ | .03^**^ | .19^**^ | -.28^**^ | .03^**^ | .06^**^ | .03^**^ | .13^**^ | .24^**^ | .29^**^ | -.05^**^ | .73^**^ |  |  |  |
| 15. ^T2^ Delayed recall | -.19^**^ | .11^**^ | .04^**^ | -.23^**^ | -.01 | .03^**^ | .00 | .06^**^ | .21^**^ | .28^**^ | -.01 | .60^**^ | .52^**^ |  |  |
| 16. ^T2^ Global cognition | -.19^**^ | .00 | .12^**^ | -.27^**^ | .05^**^ | .02 | .02^*^ | .13^**^ | .25^**^ | .33^**^ | -.05^**^ | .48^**^ | .59^**^ | .77^**^ |  |
| 17. Months between T_1_ and T_2_ | -.03^**^ | .04^**^ | -.07^**^ | .05^**^ | .13^**^ | -.02^*^ | -.04^**^ | -.08^**^ | .06^**^ | -.08^**^ | .03^**^ | -.06^**^ | -.09^**^ | -.02^*^ | .02^*^ |

*Note.* ^*^*p* ≤. 05. ^**^*p* ≤ 0.1.

**Table S3**Cross-sectional sample (imputed and unweighted): Regression analyses on delayed recall and global cognition.

|  | Delayed Memory | Global Cognition | |
| --- | --- | --- | --- |
| Predictors | Estimate (95% CI) | Estimate (95% CI) | |
| Intercept | 6.52 (3.24, 9.79)^**^ | 15.35 (11.77, 18.94)^**^ | |
| Age | -0.05 (-0.08, -0.01)^**^ | -0.03 (-0.07, 0.01) | |
| Female sex *(ref. male sex)* | 0.43 (0.12, 0.75)^*^ | 0.65 (0.30, 0.99)^**^ | |
| Education |  |  | |
| Medium *(ref. low)* | 0.06 (-0.12, 0.24) | 0.24 (0.04, 0.44)^*^ | |
| High *(ref. low/medium)* | 0.10 (-0.02, 0.22) | 0.28 (0.15, 0.41)^**^ | |
| Private housing | -0.32 (-1.02, 0.39) | -0.75 (-1.52, 0.02) | |
| Marital Status |  |  | |
| Widowed *(ref. single/divorced)* | -0.07 (-0.36, 0.21) | 0.13 (-0.19, 0.44) | |
| Married/partnered *(ref. single/divorced/widowed)* | -0.04 (-0.17, 0.08) | 0.05 (-0.09, 0.19) | |
| Close network size | 0.03 (-0.07, 0.13) | 0.03 (-0.08, 0.14) | |
| Overall network size | 0.03 (0.00, 0.05)^*^ | 0.04 (0.01, 0.06)^**^ | |
| Contact frequency | -0.02 (-0.22, 0.17) | -0.14 (-0.35, 0.07) | |
| Leisure engagement | 0.47 (0.21, 0.72)^**^ | 0.45 (0.17, 0.73)^**^ | |
| Loneliness |  |  | |
| Never *(ref. sometimes/often/always)* | 0.07 (-0.41, 0.56) | 0.17 (-0.36, 0.70) | |
| Sometimes *(ref. often/always)* | 0.10 (-0.59, 0.79) | 0.52 (-0.24, 1.27) | |
| Often *(ref. always)* | -0.13 (-1.37, 1.11) | -0.58 (-1.94, 0.77) | |
| Functional ability | 0.41 (0.10, 0.73)^**^ | 0.97 (0.63, 1.32)^**^ | |
| Depressive symptoms | 0.09 (-0.06, 0.24) | 0.06 (-0.10, 0.22) | |
| R-Squared | 0.07 | 0.12 | |
| N | 1207 | 1207 | |
| *Note.* ^*^*p* ≤ .05, ^**^*p* ≤ .01. | | |  |

**Table S4**

Panel sample (imputed and unweighted): Regression analyses on delayed recall and global cognition.

|  | ^T2^ Delayed Memory | ^T2^ Global Cognition | |
| --- | --- | --- | --- |
| Predictors | Estimate (95% CI) | Estimate (95% CI) | |
| Intercept | 3.03 (-1.33, 7.39) | 8.07 (1.80, 14.34)^**^ | |
| ^T1^ Performance | 0.55 (0.48, 0.62)^**^ | 0.64 (0.55, 0.74)^**^ | |
| Months between T_1_ and T_2_ | 0.04 (-0.03, 0.10) | 0.08 (-0.02, 0.17) | |
| ^T1^ Age | -0.04 (-0.08, -0.00) | -0.08 (-0.14, -0.02)^*^ | |
| Female sex *(ref. male sex)* | 0.46 (0.08, 0.84)^*^ | 0.46 (-0.07, 1.00) | |
| Education |  |  | |
| Medium *(ref. low)* | -0.01 (-0.23, 0.22) | 0.13 (-0.19, 0.45) | |
| High *(ref. low/medium)* | 0.11 (-0.03, 0.24) | 0.15 (-0.04, 0.34) | |
| ^T1^ Private housing | -1.10 (-1.91, -0.29)^**^ | -0.90 (-2.05, 0.26) | |
| ^T1^ Marital Status |  |  | |
| Widowed *(ref. single/divorced)* | 0.01 (-0.33, 0.35) | -0.14 (-0.62, 0.35) | |
| Married/partnered *(ref. single/divorced/widowed)* | 0.00 (-0.15, 0.16) | -0.01 (-0.23, 0.20) | |
| ^T1^ Close network size | -0.01 (-0.13, 0.10) | -0.12 (-0.29, 0.04) | |
| ^T1^ Overall network size | 0.01 (-0.02, 0.04) | 0.02 (-0.02, 0.07) | |
| ^T1^ Contact frequency | 0.01 (-0.22, 0.24) | 0.18 (-0.15, 0.51) | |
| ^T1^ Leisure engagement | 0.12 (-0.19, 0.44) | 0.25 (-0.20, 0.70) | |
| ^T1^ Loneliness |  |  | |
| Never *(ref. sometimes/often/always)* | -0.26 (-0.86, 0.35) | -0.11 (-0.96, 0.74) | |
| Sometimes *(ref. often/always)* | -0.27 (-1.16, 0.62) | -0.11 (-1.36, 1.14) | |
| Often *(ref. always)* | 0.47 (-1.16, 2.10) | -0.51 (-2.79, 1.78) | |
| ^T1^ Functional ability | 0.60 (0.18, 1.01)^**^ | 0.62 (-0.02, 1.23)^*^ | |
| ^T1^ Depressive symptoms | 0.10 (-0.09, 0.29) | 0.04 (-0.22, 0.30) | |
| R-Squared | 0.37 | 0.34 | |
| N | 639 | 625 | |
| *Note.* ^*^*p* ≤ .05, ^**^*p* ≤ .01. | | |  |

**Table S5**Cross-sectional sample (unimputed and unweighted): Regression analyses on delayed recall and global cognition.

|  | Delayed Memory | Global Cognition |
| --- | --- | --- |
| Predictors | Estimate (95% CI) | Estimate (95% CI) |
| Intercept | 5.44 (1.97 – 8.91)^**^ | 15.40 (11.57, 19.23)^**^ |
| Age | -0.04 (-0.07 – 0.00) | -0.03 (-0.07, 0.01) |
| Female sex *(ref. male sex)* | 0.45 (0.12 – 0.78)^**^ | 0.56 (0.20, 0.93)^**^ |
| Education |  |  |
| Medium *(ref. low)* | 0.01 (-0.18 – 0.20) | 0.16 (-0.05, 0.37) |
| High *(ref. low/medium)* | 0.05 (-0.07 – 0.17) | 0.25 (0.11, 0.38)^**^ |
| Private housing | -0.64 (-1.39 – 0.11) | -0.85 (-1.68, -0.03)^*^ |
| Marital Status |  |  |
| Widowed *(ref. single/divorced)* | -0.08 (-0.38, 0.22) | 0.00 (-0.33, 0.33) |
| Married/partnered *(ref. single/divorced/widowed)* | -0.08 (-0.22, 0.05) | 0.01 (-0.14, 0.16) |
| Close network size | 0.05 (-0.05, 0.15) | 0.03 (-0.08, 0.15) |
| Overall network size | 0.03 (0.00, 0.05)^*^ | 0.04 (0.01, 0.06)^*^ |
| Contact frequency | -0.09 (-0.29, 0.11) | -0.13 (-0.35, 0.09) |
| Leisure engagement | 0.40 (0.13, 0.67)^**^ | 0.29 (-0.01, 0.59) |
| Loneliness |  |  |
| Never *(ref. sometimes/often/always)* | 0.21 (-0.31 – 0.72) | 0.08 (-0.49, 0.65) |
| Sometimes *(ref. often/always)* | 0.30 (-0.44 – 1.03) | 0.43 (-0.38, 1.24) |
| Often *(ref. always)* | -0.63 (-1.95 – 0.70) | -1.25 (-2.71, 0.22) |
| Functional ability | 0.59 (0.25 – 0.92)^**^ | 1.12 (0.75, 1.49)^**^ |
| Depressive symptoms | 0.08 (-0.07, 0.23) | 0.02 (-0.14, 0.18) |
| R-Squared | 0.07 | 0.11 |
| N | 1050 | 1050 |
| *Note.* ^*^*p* ≤ .05, ^**^*p* ≤ .01. | | |

**Table S6**

Panel sample (unimputed and unweighted): Regression analyses on delayed recall and global cognition.

|  | ^T2^ Delayed Memory | ^T2^ Global Cognition |
| --- | --- | --- |
| Predictors | Estimate (95% CI) | Estimate (95% CI) |
| Intercept | 4.12 (-0.43, 8.68) | 11.00 (4.35, 17.65)^**^ |
| ^T1^ Performance | 0.58 (0.51, 0.66)^**^ | 0.62 (0.52, 0.72)^**^ |
| Months between T_1_ and T_2_ | 0.03 (-0.04, 0.10) | 0.06 (-0.04, 0.16) |
| ^T1^ Age | -0.05 (-0.09, -0.01)^*^ | -0.09 (-0.16, -0.03)^*^ |
| Female sex *(ref. male sex)* | 0.49 (0.10, 0.89)^*^ | 0.57 (0.01, 1.13)^*^ |
| Education |  |  |
| Medium *(ref. low)* | -0.08 (-0.31, 0.15) | 0.09 (-0.24, 0.42) |
| High *(ref. low/medium)* | 0.12 (-0.02, 0.26) | 0.17 (-0.03, 0.37) |
| ^T1^ Private housing | -1.15 (-1.99, -0.32)^**^ | -1.19 (-2.40, 0.02) |
| ^T1^ Marital Status |  |  |
| Widowed *(ref. single/divorced)* | -0.11 (-0.47, 0.26) | -0.14 (-0.66, 0.38) |
| Married/partnered *(ref. single/divorced/widowed)* | -0.04 (-0.21, 0.12) | 0.00 (-0.23, 0.23) |
| ^T1^ Close network size | -0.02 (-0.14, 0.10) | -0.15 (-0.32, 0.03) |
| ^T1^ Overall network size | 0.01 (-0.02, 0.04) | 0.03 (-0.02, 0.07) |
| ^T1^ Contact frequency | 0.03 (-0.20, 0.27) | 0.20 (-0.14, 0.54) |
| ^T1^ Leisure engagement | 0.05 (-0.28, 0.38) | 0.17 (-0.30, 0.63) |
| ^T1^ Loneliness |  |  |
| Never *(ref. sometimes/often/always)* | -0.22 (-0.84, 0.41) | -0.32 (-1.21, 0.57) |
| Sometimes *(ref. often/always)* | -0.39 (-1.30, 0.53) | -0.19 (-1.48, 1.10) |
| Often *(ref. always)* | 0.44 (-1.22, 2.11) | -1.06 (-3.43, 1.30) |
| ^T1^ Functional ability | 0.42 (-0.02, 0.87) | 0.36 (-0.30, 1.01) |
| ^T1^ Depressive symptoms | 0.09 (-0.09, 0.27) | -0.02 (-0.28, 0.24) |
| R-Squared | 0.36 | 0.30 |
| N | 579 | 568 |
| *Note.* ^*^*p* ≤ .05, ^**^*p* ≤ .01. | | |

**Table S7**

Cross-sectional sample (imputed and weighted): Regression analyses on delayed recall and global cognition excluding participants with mild cognitive impairment.

|  | Delayed Memory | Global Cognition |
| --- | --- | --- |
| Predictors | Estimate (95% CI) | Estimate (95% CI) |
| Intercept | 10.24 (6.67, 13.81)^**^ | 17.14 (14.19, 20.09)^**^ |
| Age | -0.07 (-0.11, -0.03)^**^ | -0.02 (-0.06, 0.01) |
| Female sex *(ref. male sex)* | 0.40 (0.07, 0.73)^*^ | 0.40 (0.14, 0.67)^**^ |
| Education |  |  |
| Medium *(ref. low)* | 0.07 (-0.11, 0.24) | 0.20 (0.05, 0.34)^**^ |
| High *(ref. low/medium)* | 0.01 (-0.11, 0.13) | 0.14 (0.05, 0.24)^**^ |
| Private housing | 0.13 (-0.56, 0.82) | -0.40 (-0.97, 0.18)^**^ |
| Marital Status |  |  |
| Widowed *(ref. single/divorced)* | -0.09 (-0.34, 0.16) | 0.02 (-0.19, 0.23) |
| Married/partnered *(ref. single/divorced/widowed)* | -0.10 (-0.22, 0.02) | -0.02 (-0.12, 0.08) |
| Close network size | 0.02 (-0.08, 0.13) | 0.03 (-0.05, 0.11) |
| Overall network size | 0.00 (-0.03, 0.02) | 0.02 (0.00, 0.04) |
| Contact frequency | 0.04 (-0.15, 0.23) | -0.10 (-0.26, 0.05) |
| Leisure engagement | 0.40 (0.14, 0.66)^**^ | 0.22 (0.00, 0.43)^*^ |
| Loneliness |  |  |
| Never *(ref. sometimes/often/always)* | 0.12 (-0.40, 0.65) | 0.13 (-0.30, 0.56) |
| Sometimes *(ref. often/always)* | -0.15 (-0.89, 0.59) | 0.22 (-0.40, 0.83) |
| Often *(ref. always)* | 0.49 (-0.83, 1.81) | -0.25 (-1.34, 0.84) |
| Functional ability | 0.03 (-0.31, 0.38) | 0.35 (0.07, 0.64)^*^ |
| Depressive symptoms | -0.01 (-0.16, 0.14) | 0.03 (-0.09, 0.15) |
| R-Squared | 0.06 | 0.07 |
| N | 979 | 979 |
| *Note.* ^*^*p* ≤ .05, ^**^*p* ≤ .01. | | |

**Table S8**

Panel sample (imputed and weighted): Regression analyses on delayed recall and global cognition excluding participants with mild cognitive impairment at first wave.

|  | ^T2^ Delayed Memory | ^T2^ Global Cognition |
| --- | --- | --- |
| Predictors | Estimate (95% CI) | Estimate (95% CI) |
| Intercept | 4.28 (-0.69, 10.33) | 11.72 (4.64, 18.80)^**^ |
| ^T1^ Performance | 0.55 (0.46, 0.64)^**^ | 0.58 (0.44, 0.71)^**^ |
| Months between T_1_ and T_2_ | 0.00 (-0.07, 0.08) | 0.05 (-0.05, 0.14) |
| ^T1^ Age | -0.06 (-0.11, 0.00)^*^ | -0.09 (-0.16, -0.02)^**^ |
| Female sex *(ref. male sex)* | 0.55 (0.10, 1.01)^*^ | 0.61 (0.05, 1.16)^*^ |
| Education |  |  |
| Medium *(ref. low)* | 0.00 (-0.24, 0.25) | 0.01 (-0.29, 0.32) |
| High *(ref. low/medium)* | 0.06 (-0.09, 0.21) | 0.13 (-0.06, 0.32) |
| ^T1^ Private housing | -1.42 (-2.33, -0.51)^**^ | -1.47 (-2.61, -0.33) |
| ^T1^ Marital Status |  |  |
| Widowed *(ref. single/divorced)* | -0.12 (-0.46, 0.22) | -0.19 (-0.62, 0.24) |
| Married/partnered *(ref. single/divorced/widowed)* | -0.04 (-0.21, 0.12) | 0.01 (-0.19, 0.21) |
| ^T1^ Close network size | -0.02 (-0.16, 0.12) | -0.11 (-0.27, 0.06) |
| ^T1^ Overall network size | 0.00 (-0.04, 0.03) | 0.02 (-0.02, 0.07) |
| ^T1^ Contact frequency | 0.02 (-0.25, 0.28) | 0.07 (-0.26, 0.40) |
| ^T1^ Leisure engagement | 0.07 (-0.29, 0.44) | -0.10 (-0.55, 0.35) |
| ^T1^ Loneliness |  |  |
| Never *(ref. sometimes/often/always)* | 0.05 (-0.75, 0.85) | 0.01 (-0.96, 0.98) |
| Sometimes *(ref. often/always)* | -0.19 (-1.35, 0.97) | -0.14 (-1.56, 1.27) |
| Often *(ref. always)* | -0.21 (-2.36, 1.95) | -0.52 (-3.14, 2.10) |
| ^T1^ Functional ability | 0.79 (0.31, 1.27)^**^ | 0.73 (0.12, 1.34)^**^ |
| ^T1^ Depressive symptoms | 0.21 (0.00, 0.42)^*^ | 0.00 (0.26, 0.26) |
| R-Squared | 0.22 | 0.32 |
| N | 560 | 560 |
| *Note.* ^*^*p* ≤ .05, ^**^*p* ≤ .01. | | |
